# Attraction effect of different colored cards on thrips *Frankliniella intonsa* in cowpea greenhouses in China

**DOI:** 10.1038/s41598-018-32035-8

**Published:** 2018-09-11

**Authors:** Liangang Mao, Yiming Chang, Fulai Yang, Lan Zhang, Yanning Zhang, Hongyun Jiang

**Affiliations:** State Key Laboratory for Biology of Plant Disease and Insect Pests, Institute of Plant Protection, Chinese Academy of Agricultural Sciences, Ministry of Agriculture and Rural Affairs of People’s Republic of China, Beijing, 100193 China

## Abstract

The flower thrips *Frankliniella intonsa* (Trybom) is one of the most economically important pests in cowpea greenhouses in China. Widespread pesticide resistance of thrips and the negative environmental effects limit the application of pesticides for thrips control. Two commercial cowpea greenhouse experiments were designed to determine the color preference of *F. intonsa* to colored cards, including white, pink, pale green, light yellow, powder blue, crimson, yellow green, deep sky blue, dark slate blue, dark orange, medium orchid, gold, and black. Clear pieces of plastic were used as the control cards. Additionally, the effects of placement height and orientation (cardinal direction) of the cards were also studied. Both greenhouse trials showed that white cards were significantly more attractive to *F. intonsa* than the other 13 card colors, followed by deep sky blue cards. White or deep sky blue cards placed low to the ground were found to be most attractive to *F. intonsa*. Orientation of the colored cards also affected the attractiveness to *F. intonsa*. The results indicate that white sticky cards were significantly more attractive to *F. intonsa* than blue cards and therefore can be recommended to monitor *F. intonsa* population densities and to control them in cowpea greenhouses as part of integrated pest management programs.

## Introduction

Cowpea (*Vigna unguiculata* (Linn.) Walp.), also known as Chinese long bean, is widely cultivated as a vegetable in China^1^. Thysanoptera (thrips), one of most economically important insect pest, regularly cause serious damage to cowpea grown in protected fields through direct feeding and virus transmission^2,3^. The flower thrips *Frankliniella intonsa* (Trybom) is one of the most important thrips species affecting the yield and market value of cowpea in China^4^.

Applying pesticides is the main method to control thrips in China. Currently, registered insecticides on cowpea for thrips control in China include acetamiprid, thiamethoxam, emamectin benzoate, spinosad, and cyantraniliprole^5^. However, pesticides efficacy is limited by the widespread resistance of thrips to most conventional insecticides and the negative environmental effects of pesticides^6–8^. In 2015, the Chinese Ministry of Agriculture introduced special measures that seek to halt the growth in the use of pesticides: the “Action to Achieve Zero Growth of Pesticide Use by 2020”^9^. With the implementation of the pesticides reduction plan, increasing attention is being paid to non-chemical methods for plant protection in China.

Blue sticky card traps are often recommended as a non-chemical method for the control of thrips in the field or greenhouse in China^10^. However, attractiveness of cards varies among thrips species and among card colors, including blue, yellow, red, white and others^8,11–14^. In one of the studies, blue, cyan and white cards were found very attractive to *F. intonsa* in a balsam pear field, but there was no significant difference in attractiveness among the three kinds of colored cards^15^. Additionally, there was no report on the color preference of *F. intonsa* in cowpea habitats.

The current study was designed to determine the color preference of *F. intonsa* in two cowpea greenhouse trials, evaluating attractiveness of cards that were white, pink, pale green, light yellow, powder blue, crimson, yellow green, deep sky blue, dark slate blue, dark orange, medium orchid, gold, and black. Clear pieces of plastic were used as control cards. Additionally, the effects of orientation (cardinal direction) and placement height of the cards was evaluated.

## Results

### Effect of card color on thrips

**Figure 1:**
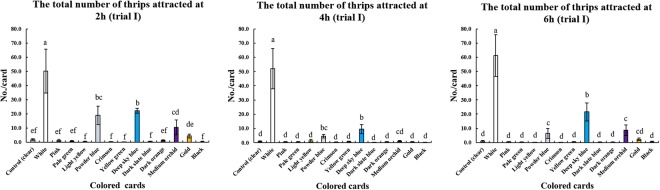
The attractiveness of different colored cards to thrips at 2 h, 4 h and 6 h (trial I) (mean ± SEM).

**Figure 2:**
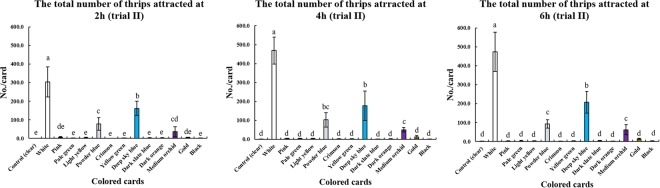
The attractiveness of different colored cards to thrips at 2 h, 4 h and 6 h (trial II) (mean ± SEM).

The numbers of thrips attracted to the cards varied among the 13 colors (Figs 1 and 2). The control (clear) cards were unattractive to thrips (less than 2 and 1 thrips per card, respectively) in both greenhouse trials (Tables S1 and S2). Among the 13 colored cards, the white cards had the strongest attractive ability, attracting up to 61 thrips per card in trial I and 474 thrips per card in trial II (Tables S1 and S2). White cards were significantly more attractive than the other color in both trials at 2 h, 4 h and 6 h after the start of the experiment (*P* = 0.05) (Figs 1 and 2).

In greenhouse trial I, the white cards had the strongest attractive ability to thrips, followed by deep sky blue, powder blue, medium orchid, gold and the other color after 2 h (Fig. 1). Furthermore, the number of thrips attracted to the deep sky blue cards was similar to the powder blue cards but was significantly higher than the other colored cards (*P* = 0.05) (Fig. 1). After 4 h, the numbers of thrips attracted to the white cards increased, while the number of thrips attracted on the deep sky blue or powder blue cards both decreased (Fig. 1). Furthermore, the number of thrips attracted to the deep sky blue cards was similar to the powder blue cards, but significantly higher than the other colors (*P* = 0.05) (Fig. 1), which all showed no significant difference from the control (clear) cards in attracting thrips (*P* = 0.05) (Fig. 1). After 6 h, white cards still showed the strongest attractive ability, followed by deep sky blue (Fig. 1), and deep sky blue cards attracted significantly more thrips than each of the other colors, except for white (*P* = 0.05) (Fig. 1).

In greenhouse trial II, white cards also had the strongest attractive ability (303 thrips per card) after 2 h, followed by deep sky blue, powder blue, medium orchid and the other colors (Fig. 2). Furthermore, the number of thrips attracted to deep sky blue cards (161 thrips per card) was significantly higher than to powder blue cards (78 thrips per card) (*P* = 0.05). In addition, the number of thrips attracted to the powder blue cards was similar to medium orchid cards, but significantly higher than the rest of the colors (*P* = 0.05) (Fig. 2). After 6 h, the numbers of thrips attracted to the white, deep sky blue, powder blue, and medium orchid cards all increased, except for the powder blue cards (Fig. 2). After 4 h, the number of thrips attracted to deep sky blue cards was similar to powder blue cards, but significantly higher the other colors (*P* = 0.05) (Fig. 2). Except for white, deep sky blue, powder blue and medium orchid, there was no significant difference between each of the other colored cards and the control (clear) cards (*P* = 0.05) (Fig. 2). After 6 h, the deep sky blue cards showed significantly stronger attractive ability than the powder blue cards (Fig. 2). In addition, the number of thrips attracted to the powder blue cards was similar the medium orchid cards but was significantly higher than the other colors (*P* = 0.05) (Fig. 2).

### Effect of card placement height on thrips

The white and deep sky blue cards, as the two most attractive colors, were selected to evaluate the effect of card placement height on thrips. The number of thrips attracted to the white or deep sky blue cards differed with card placement height (Table 1). For the white cards, the number of thrips attracted at the low position was always significantly higher than that at the middle or high position (*P* = 0.05), except for the middle position in trial I at 6 h. For the deep sky blue cards, the number of thrips attracted at the low position was also always significantly higher than at the middle or high position (*P* = 0.05), except for the middle position in trial I at 4 h. In both trials, whether the cards were white or deep sky blue, there was no significant difference between the middle position and the high position at 2 h, 4 h and 6 h (*P* = 0.05) (Table 1).

**Table 1:** The attractiveness of two selected colored cards with different height in trials I and II. (Values are expressed as the mean ± SEM of four replicates, with 2 cards per replicate, *P* < 0.05).

Trials	Color	Height	Total number of thrips per card		
			2 h	4 h	6 h
Trial I	White	High	34.9 ± 4.9b	30.6 ± 4.0b	29.8 ± 11.2b
		Middle	60.5 ± 14.7b	84.3 ± 30.1b	97.5 ± 40.9ab
		Low	131.1 ± 23.3a	155.5 ± 26.7a	179.0 ± 41.8a
	Deep sky blue	High	15.1 ± 5.5b	7.8 ± 5.7b	15.6 ± 10.1b
		Middle	33.0 ± 5.1b	15.0 ± 7.7ab	36.9 ± 14.1b
		Low	64.0 ± 10.2a	37.3 ± 15.5a	70.4 ± 21.9a
Trial II	White	High	146.6 ± 50.7b	229.5 ± 54.3b	274.9 ± 77.7b
		Middle	171.3 ± 55.4b	289.9 ± 41.3b	291.3 ± 56.8b
		Low	593.8 ± 137.6a	887.8 ± 128.2a	856.8 ± 197.7a
	Deep sky blue	High	95.8 ± 20.7b	71.8 ± 38.1b	96.5 ± 25.1b
		Middle	81.5 ± 25.5b	132.8 ± 58.6b	146.8 ± 42.8b
		Low	307.0 ± 76.3a	327.6 ± 137.1a	378.9 ± 105.7a

### Effect of card cardinal direction on thrips

The white and deep sky blue cards, as the two most attractive colors, were also selected to evaluate the effect of card orientation (cardinal direction) on thrips. The number of thrips attracted to the white or deep sky blue cards varied with card orientation (Table 2). For white cards in trial I at 2 h, there were more thrips attracted to the side facing to the south or east than to the side facing north or west (*P* = 0.05). However, the significant difference disappeared at 4 h or 6 h. There was no significant difference between cardinal directions in trial II (*P* = 0.05), except for the side facing east at 4 h (Table 2). For the deep sky blue cards in trial I at 2 and 4 h, there were more thrips attracted on the side facing to the east than to the side facing west (*P* = 0.05). However, there were more thrips attracted to the side facing south than to other cardinal directions in trial II (*P* = 0.05).

**Table 2:** The attractiveness of two selected colored cards with different orientations to thrips in trials I and II. (Values are expressed as the mean ± SEM of four replicates, with 6 sides per replicate, *P* < 0.05).

Trials	Color	Orientation	Total number of thrips per side		
			2 h	4 h	6 h
Trial I	White	East	37.8 ± 11.7a	35.9 ± 9.0a	41.8 ± 16.3a
		West	10.4 ± 5.2b	17.3 ± 3.0a	28.9 ± 20.9a
		South	38.8 ± 7.6a	37.5 ± 13.3a	20.2 ± 6.2a
		North	13.3 ± 6.8b	21.9 ± 10.6a	31.6 ± 13.8a
	Deep sky blue	East	19.3 ± 5.9a	8.9 ± 3.3a	12.3 ± 4.7a
		West	2.3 ± 0.6b	3.3 ± 1.9b	10.8 ± 5.2a
		South	8.8 ± 5.3ab	5.8 ± 2.3ab	14.3 ± 5.9a
		North	13.9 ± 3.3ab	4.4 ± 1.9b	5.8 ± 2.2a
Trial II	White	East	37.4 ± 8.9a	26.8 ± 7.4b	19.4 ± 9.0a
		West	37.6 ± 21.0a	111.7 ± 47.8ab	157.3 ± 57.2a
		South	170.5 ± 80.4a	235.3 ± 89.0a	189.1 ± 91.0a
		North	63.9 ± 15.1a	99.9 ± 48.3ab	112.0 ± 54.6a
	Deep sky blue	East	15.8 ± 7.1b	25.8 ± 14.1b	23.8 ± 5.5b
		West	17.3 ± 6.5b	34.8 ± 23.3b	49.3 ± 17.9b
		South	107.1 ± 24.3a	77.2 ± 20.7a	108.8 ± 25.3a
		North	22.3 ± 9.2b	42.6 ± 20.5b	28.9 ± 18.1b

As one card always has two sides, the number of thrips on the two sides was summed for further analysis, for example, east-west and south-north (Table S3). For white cards, there was no significant difference in the number of thrips attracted to east-west versus south-north in both greenhouse trials, except for trial II at 2 h. For the deep sky blue cards, there was also no significant difference in trial I, but the number of thrips attracted to south-north facing cards was significantly higher than east-west facing cards in trial II at 2 h, 4 h and 6 h (*P* = 0.05) (Table S3).

## Discussion

Blue was reported to be the most attractive color to *F. intonsa* in a balsam pear field^15^ and a strawberry field^16^, followed in attractiveness by white, but there was no significant difference between blue and white in the balsam pear field^15^. However, white cards were found here to have a significantly stronger attractive ability than blue cards (deep sky blue or powder blue) for *F. intonsa* in our two cowpea greenhouse trials. Furthermore, the attractiveness to *F. intonsa* varied among different blue colors in the two trials, for example, deep sky blue, powder blue and dark slate blue (Figs 1 and 2). It may be possible to find another blue color except for the three blue colors tested here (deep sky blue, powder blue and dark slate blue) that was as attractive as white to *F. intonsa*. However, the greenhouse trials here indicated that white is the best color for attracting *F. intonsa*, rather than blue or yellow cards in cowpea greenhouses.

In our two cowpea greenhouse trials, the cards at the low position were usually found to attract more thrips than at the middle or high positions (*P* = 0.05) (Table 2), which could be attributed to thrips behavior of entering the soil for pupation^17^. Furthermore, both of the two greenhouse trials were carried out at noon, so thrips perhaps gathered at lower positions of the cowpea plants to escape the strong sunlight due to the concealment behavior^18^. This may be another reason for attracting more thrips at the low position.

In our two cowpea greenhouse trials, the main species of thrips was identified to be *F. intonsa* in each case. Indeed, there are many thrips species infesting different crops in the field, but different species of thrips are attracted to different colors^14,19^. To effectively monitor thrips population densities and control different thrips species, first testing different colored cards to screen for optimal color is necessary before applying colored cards in the field. To enhance the pest control of *F. intonsa*, pheromone could be used in combination with white sticky cards^20^.

Additionally, in order to successfully control *F. intonsa* with IPM, other biological control agents should also be considered^21^, for example, silver stripe mulching film^22^, predatory mites^23^, and entomopathogenic fungi^18,24^. No beneficial insects were present during our two greenhouse trials, so the potential effect of colored cards on beneficial insects (e.g., lacewings) should also be considered in future work^25^.

In summary, white cards showed the strongest attraction ability for *F. intonsa* among the 13 different colored cards in each of two cowpea greenhouse trials. However, more detailed work on potential combinations with pheromones or other biological agents (e.g., predatory mites, entomopathogenic fungi or others) are necessary before white sticky cards can be recommended as an efficient method to control *F. intonsa* in cowpea greenhouses in China.

## Materials and Methods

### Greenhouse trial site

Two greenhouse trials were conducted in two commercial cowpea greenhouses in Fangshan, Beijing (trial I, N39°38′19.0″ E116°01′32.8″; trial II, N39°38′20.4″ E116°01′2.6″). There was no pesticide application for thrips control during the trials.

### Colored card selection

Thirteen colors were selected from the Encycolorpedia (http://encycolorpedia.com/) for making colored cards (white, pink, pale green, light yellow, powder blue, crimson, yellow green, deep sky blue, dark slate blue, dark orange, medium orchid, gold, and black). Cards of all of the above colors were printed on A4-size paper (297 mm × 210 mm) and sealed with two pieces of clear laminating film. Clear pieces of plastic with the same size were used as the untreated control. The RGB values of the above cards are listed in Table 3.

**Table 3:** The tested card colors and the corresponding R.G.B. values.

No.	Color	R.G.B. values^1^
/	Control (clear)	/
1	White	255 255 255
2	Pink	255 192 203
3	Pale green	152 251 152
4	Light yellow	255 255 224
5	Powder blue	176 224 230
6	Crimson	220 20 60
7	Yellow green	154 205 50
8	Deep sky blue	0 191 255
9	Dark slate blue	72 61 139
10	Dark orange	255 140 0
11	Medium orchid	186 85 211
12	Gold	255 215 0
13	Black	0 0 0

Note: ^1^The RGB values of the colors are taken from the Encycolorpedia (http://encycolorpedia.com/).

### Experimental design

**Figure 3:**
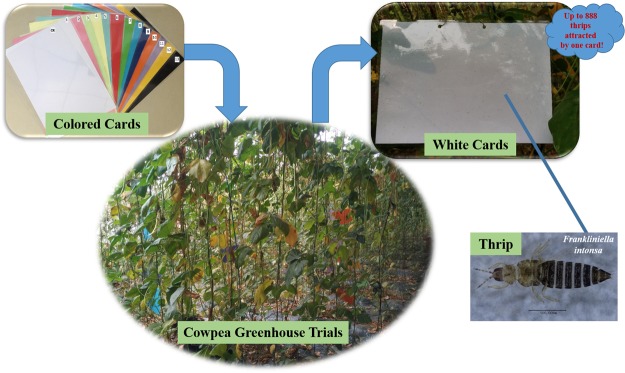
The colored cards field trials diagram.

The thirteen colored cards and clear controls were employed as randomized blocks with four replicates each in trials I and II in July, 2017. Two cardinal direction (east-west, and south-north) and three placement heights (0 cm, 80 cm, and 160 cm) were studied at the same time. Every plot area was designed in 15 m^2^ with 6 same colored cards. Six cards of each color were employed at each of six positions (east-west/0 cm, east-west/80 cm, east-west/160 cm, south-north/0 cm, south-north/80 cm, south-north/160 cm) and were placed randomly in each plot. The number of thrips attracted by both sides of the cards were counted at 2 h, 4 h and 6 h after the placement of cards. The whole experimental design was briefly descripted in Fig. 3, and all of the photos in Fig. 3 were taken in the greenhouse trials and the laboratory by the authors.

### Statistical analysis

The average number of thrips per card, including both sides, for each color in each plot were used to evaluate the attraction effect of the different colors. The average number of thrips per card for each height by two different cardinal directions (east-west, and south-north) in each plot were used to evaluate the attraction effect of card height. The average number of thrips attracted per side of each card for each of the four cardinal directions and by three different placement heights (0 cm, 80 cm, and 160 cm) in each plot were used to evaluate the attraction effect of card orientation.

All of the treatments were performed with four plot replicates at each of the two trials. Data for thrips populations were transformed as necessary for statistical analyses (square root transformations for small numbers [<100] and log10 for large numbers [>100]), but all data are reported as untransformed values. Data were analyzed using ANOVA with SPSS (version 22.0 for Windows). Significant differences among means were tested with Fisher’s LSD at *P* = 0.05^26,27^.

## Electronic supplementary material

Supplementary Information
