# Supplementary material for: Attraction effect of different colored cards on thrips Frankliniella intonsa in cowpea greenhouses in China
Source: Sci Rep. 2018 Sep 11;8:13603. doi: 10.1038/s41598-018-32035-8 (PMC6133937; doi:10.1038/s41598-018-32035-8)
Supplement: Supplementary file 1 — Supplementary Information [file 41598_2018_32035_MOESM1_ESM.docx]

**Supplementary information**

**Attraction effect of different colored cards on thrips *Frankliniella intonsa* in cowpea greenhouses in China.**

Liangang Mao, Yiming Chang, Fulai Yang, Lan Zhang, Yanning Zhang & Hongyun Jiang^*^

State Key Laboratory for Biology of Plant Disease and Insect Pests, Institute of Plant Protection, Chinese Academy of Agricultural Sciences, Ministry of Agriculture and Rural Affairs of People’s Republic of China, Beijing 100193.

Correspondence and requests for materials should be addressed to H.J. (Phone: +8610-62893622; E-mail: ptnpc@vip.163.com)

This supplementary information file contains 3 Supplemental tables.

**Table S1** The attractiveness of different cards colors to thrips at 2 h, 4 h and 6 h (trial I). (Values are expressed as the mean±SEM of four replicates, with 6 cards per replicate, *P*<0.05)

| **Color** | **Total number of thrips per card** | | |
| --- | --- | --- | --- |
|  | **2 h** | **4 h** | **6 h** |
| Control (clear) | 1.7±0.8ef | 0.8±0.4d | 0.8±0.5d |
| White | 50.2±15.4a | 52.0±14.1a | 61.2±14.8a |
| Pink | 1.3±0.8ef | 0.5±0.3d | 0.5±0.1d |
| Pale green | 0.7±0.4ef | 0.5±0.2d | 0.3±0.2d |
| Light yellow | 0.5±0.3f | 1.2±1.0d | 0.1±0.1d |
| Powder blue | 19.0±6.4bc | 4.6±1.2bc | 6.4±3.6c |
| Crimson | 0.3±0.3f | 0.3±0.3d | 0.0±0.1d |
| Yellow green | 0.2±0.1f | 0.5±0.6d | 0.4±0.4d |
| Deep sky blue | 22.2±1.7b | 9.5±3.2b | 21.5±6.4b |
| Dark slate blue | 0.4±0.3f | 0.1±0.1d | 0.2±0.1d |
| Dark orange | 1.2±0.5ef | 0.6±0.3d | 0.2±0.1d |
| Medium orchid | 10.5±5.2cd | 1.1±0.4cd | 8.6±3.7c |
| Gold | 4.4±1.4de | 0.5±0.3d | 2.3±0.8cd |
| Black | 0.3±0.3f | 0.2±0.2d | 0.5±0.3d |

**Table S2** The attractiveness of different colored cards to thrips at 2 h, 4 h and 6 h (trial II). (Values are expressed as the mean±SEM of four replicates, with 6 cards per replicate, *P*<0.05)

| **Color** | **Total number of thrips per card** | | |
| --- | --- | --- | --- |
|  | **2 h** | **4 h** | **6 h** |
| Control (clear) | 0.0±0.0e | 0.1±0.1d | 0.0±0.1d |
| White | 303.9±80.2a | 469.0±71.1a | 474.3±103.3a |
| Pink | 6.6±4.4de | 4.5±1.7d | 2.9±2.0d |
| Pale green | 1.1±0.9e | 4.0±1.6d | 3.1±2.2d |
| Light yellow | 3.3±3.0e | 3.2±2.9d | 2.5±2.1d |
| Powder blue | 78.2±33.6c | 102.9±38.3bc | 92.8±22.5c |
| Crimson | 0.0±0.0e | 1.5±1.5d | 1.0±0.4d |
| Yellow green | 2.2±1.2e | 2.0±1.3d | 2.2±1.7d |
| Deep sky blue | 161.4±39.0b | 177.4±76.9b | 207.4±57.1b |
| Dark slate blue | 2.6±2.3e | 1.1±1.0d | 3.3±2.6d |
| Dark orange | 2.4±1.5e | 2.2±1.1d | 1.0±0.9d |
| Medium orchid | 35.9±27.1cd | 50.3±12.0c | 62.4±26.1c |
| Gold | 5.9±0.9de | 12.0±7.5d | 13.0±4.5d |
| Black | 1.0±0.8e | 0.8±0.8d | 2.0±2.0d |

**Table S3** The attractiveness of two selected colored cards with different orientations to thrips in trials I and II. (Values are expressed as the mean±SEM of four replicates, with 6 cards per replicate, *P*<0.05)

| **Trials** | **Color** | **Orientation** | **Total number of per card** | | |
| --- | --- | --- | --- | --- | --- |
|  |  |  | **2 h** | **4 h** | **6 h** |
| Trial I | White | East-west | 48.3±16.8a | 53.3±9.0a | 70.7±20.2a |
|  |  | South-north | 52.2±13.9a | 59.4±21.0a | 51.8±15.5a |
|  | Deep sky blue | East-west | 21.6±5.7a | 12.3±4.5a | 23.0±6.3a |
|  |  | South-north | 22.8±4.8a | 10.3±4.2a | 20.1±6.8a |
| Trial II | White | East-west | 75.0±22.9b | 138.4±46.6a | 176.8±63.2a |
|  |  | South-north | 234.4±66.3a | 335.2±93.4a | 301.1±90.8a |
|  | Deep sky blue | East-west | 33.1±12.7b | 60.7±37.3b | 73.1±19.9b |
|  |  | South-north | 129.4±27.3a | 119.8±40.6a | 137.7±40.1a |
